# Nanostructured Bubble Thin Films—From Simple Fabrication to Scalable Applications: A Review

**DOI:** 10.3390/nano15110868

**Published:** 2025-06-04

**Authors:** Naif Ahmed Alshehri

**Affiliations:** Department of Physics, College of Science, Al-Baha University, Al-Baha 65779, Saudi Arabia; nalshehri@bu.edu.sa

**Keywords:** nanomaterials, thin films, bubbles, polymers, surfactants

## Abstract

Several applications for nanotechnology necessitate the assembly of nanomaterials over large areas with precise orientation and density. Some techniques, such as Langmuir–Blodgett, contact printing, electric field directed assembly, and flow-assisted alignment, have been used to meet such a requirement. However, it remains uncertain whether these techniques can be used for scaling up nanomaterial thin films onto large solid and flexible substrates. Accordingly, this review paper addresses such an issue by reviewing two recent flexible and scalable methods: blown bubble films (BBFs) and the bubble deposition method (BDM). It specifically offers a comprehensive account of these two bubble thin film methods along with their recent applications. It also discusses how nanomaterial thin films are made to fabricate devices. It finally provides some recommendations for further research and applications.

## 1. Introduction

Nanotechnology is a combination of science and engineering dealing with atoms and molecules of materials at the nanoscale, which have several types based on their dimensions, including zero, one, and two [1,2,3]. Such nanomaterials have unique physical, chemical, and biological [4,5] properties, such as a high surface-area-to-volume ratio [6,7], quantum confinement [8,9], reactivity [10,11,12], stability [13], biocompatibility [14], and immunogenicity [15,16]. Thus, they have a wide range of applications across different fields, such as medicine [17,18,19], environmental [20,21,22], food technology [23,24], and cosmetics [25]. Amongst all these applications, advanced energy [26,27], electronic [28,29], and optical fields [30,31] use nanomaterials for driving innovation, improving efficiency, and addressing global challenges related to sustainability and energy consumption. The unique physical properties of nanomaterials play a key role in building blocks for advanced energy, electronic, and optical applications, such as transistors [32,33], semiconductors [34], flexible electronics [35], solar cells [36,37], batteries [38,39], and supercapacitors [40,41]. However, the controlled organization of those small nanomaterial structures has an important role to play in those real-life applications [42]. Thus, they must be assembled onto solid substrates using methods that lead to an integrated system. To elaborate, some methods have been developed to assemble nanomaterials, such as Langmuir–Blodgett [43], contact printing [44], electric field directed assembly [45], and flow-assisted alignment [46]. However, one important issue that remains unaddressed is associated with the implementation of these approaches for large-scale applications [42]. There are also other issues around these methods, such as high cost and unreliability, slow operation speed, poor alignment, low density, and lack of uniformity. These issues have been considered by two recent methods, namely bubble blown films (BBFs) and the bubble deposition method (BDM). Thus, the current review paper is concerned with providing a detailed account of these two methods, aiming to offer useful insights into the strengths and limitations associated with bubble thin film production and nanomaterial thin film fabrication.

BBFs and the BDM rely on using different types of surfactants and polymers to generate bubbles for forming nanomaterial thin films. To elaborate, the BBFs’ basic approach involves the preparation of a homogeneous, stable, and controlled solution consisting of a mixture of nanomaterials and polymers. Such a polymer suspension is blown at a controlled direction and speed using air to form a bubble, which is transferred onto a substrate to gain well-defined nanomaterial-incorporated thin films. The basic idea of this method comes from the blown film extrusion process for manufacturing a plastic film in large quantities [47,48]. It forms a balloon by extruding a molten polymer which then collapses to form a flat film. The BDM, in contrast, is a method that controls the chemical potential of nanomaterials molecules to be inserted within newton black films (NBFs) or common black films (CBFs), which are then self-assembled onto a substrate [49]. These two films are made up of thin surfactant films which consist of a layer of water trapped between two layers of amphiphilic molecules, with their hydrophobic parts facing outward. The CBFs have a rather large water core compared to that of NBFs, which is much thinner, holding a small amount of residual hydration water in the core after maximum drainage [50,51]. Nanomaterials are then inserted within surfactant walls due to the two different physical processes (diffusion or electrostatic attraction) between the nanomaterials and surfactant walls. These two methods will be discussed in greater depth below.

## 2. Bubble Blown Film (BBF) Method

The bubble blown film (BBF) method is simply a method where a bubble should be formed to make nanomaterial thin films. G.Y. et al. (2007) conducted a study to generate nanomaterial thin films with a high density and alignment over a large area on silicon and flexible plastic substrates using BBFs [42]. These films consisted of a polymer solution (epoxy resin), involving nanomaterials such as carbon nanotubes, cadmium sulfide nanowires, and silicon nanowires, which were blown by air to form a bubble to be deposited onto the substrates. Wu. et al. (2014) carried out a study employing BBFs [52] with some modifications, such as using poly(methylmethacrylate) (PMMA) polymer instead of epoxy resin polymer. Such a study produced a single and a double layer of Tellurium Nanowires (Te NWs) and buckled Te nanosprings, which were then used for the synthesis of photodetectors and gas sensors devices with high performance [52]. Wu. et al. (2015) also undertook another study to develop the BBFs’ method using the same experimental techniques but with another type of polymer: additive (F-127). Such a polymer allowed them to produce a hybrid structure containing reduced graphene oxide (RGO) adhered to a carbon film [53].

It has been observed in the literature that many researchers preferred the PMMA polymer matrix to blow bubbles containing nanomaterials to be deposited onto solid and flexible substrates and used for different applications. (More information about this will be provided at an appropriate point below.) For example, Wu. et al., in 2015 [54], fabricated a self-assembled carbon nanotube (CNT) array, which was then transformed into CNT-graphene by thermal annealing onto a copper substrate (Cu). Such a film showed an improvement in electrical conductivity and structural integrity. Wu. et al., in 2015 [54], also undertook a study to assemble graphene oxide (GO) into Cu substrate, which they annealed to obtain reduced GO (carbon hybrid structure). Such a structure demonstrated potential applications in thin film electronic devices and photovoltaic applications. Wu. et al., in 2016 [55], also found that a sausage-like graphene structure, which involved multilayer graphene nanotubes and copper nanowires, can be fabricated and used for heterojunction electronic device applications [55].

**Figure 1:**
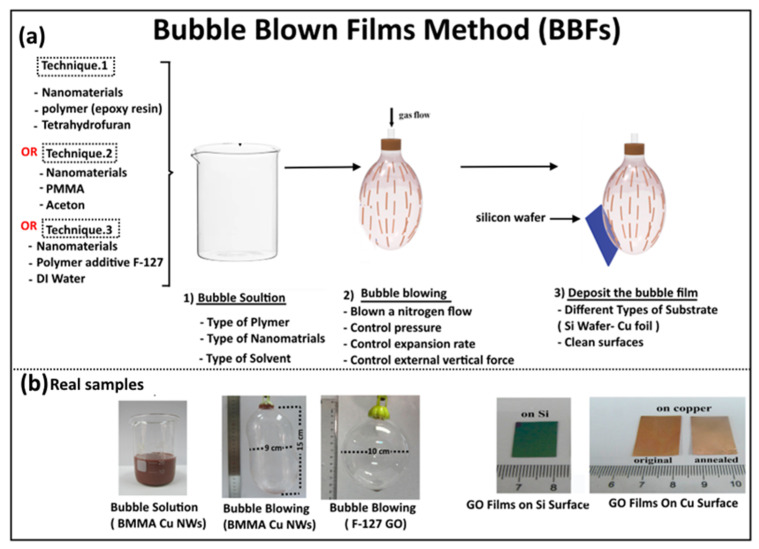
Schematic representation of bubble blown films (BBFs), illustrating the key stages to perform the experimental procedure. (**a**) The three main stages toward making thin films, which are (1) bubble solution, (2) bubble blowing, and (3) deposition of the bubble thin film. The bubble solution can be formed with three main techniques summarized on up left side of the figure. Adapted with permission from Ref. [55]. 2016, American Chemical Society. (**b**) Some of the real images taken during the laboratory experiment, illustrating the bubble solution stage (bubble solution of PMMA-Cu nanowires), the bubble-blowing stage (bubble blown films with large diameters and height up to 15 cm for Cu NWs and GO), and the deposition of the bubble film films (graphene oxide (GO) films onto top silicon and copper substates) Adapted with permission from Ref [53]. 2015, American Chemical Society. Adapted with permission from Ref [55]. 2016, American Chemical Society.

**Figure 2:**
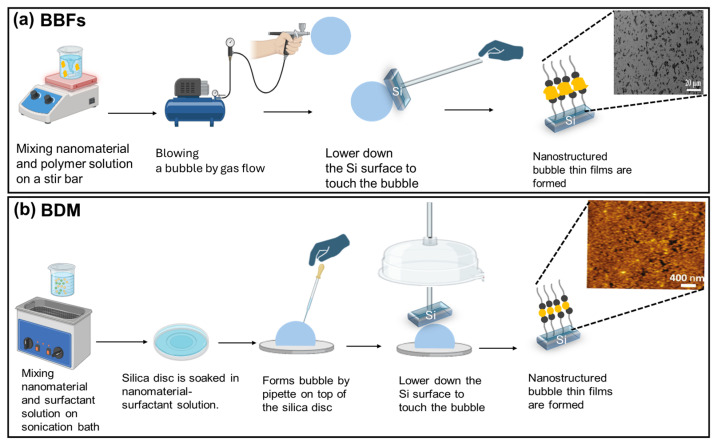
Schematic representation of bubble blown films (BBFs), and bubble deposition method (BDM), illustrating the key steps to make nanostructured bubble thin films, showing images for comparing their alignment, and density. (**a**) The BBFs’ method steps started by (1) mixing nanomaterial and polymer solution on a stir bar, (2) blowing a bubble by a gas flow, (3) lowering the Si surface to touch the bubble, (4) depositing the nanostructured bubble thin films. The SEM image of graphene oxide sheets film. Adapted with permission from Ref [53]. 2016, American Chemical Society. (**b**) The BDM steps started by (1) mixing nanomaterial and surfactant solution on a sonication bath, (2) soaking the silica disc in nanomaterial surfactant solution, (3) forming a bubble by a pipette on top of the silica disc, (4) lowering the Si surface until it touched the bubble surface, (5) depositing the nanostructured bubble thin films. The AFM topography image of a silica nanoparticles film deposited onto a silicon substrate. Adapted with permission from Ref [56]. 2010, American Chemical Society.

The relevant literature indicates that there are three techniques for forming a bubble. The first technique was developed by Yu et al. in 2007 [42], when they used a bubble containing a mixture of polymer (epoxy resin) and nanomaterial, which was then deposited into a solid or a plastic substrate. The steps used to generate the bubble are illustrated in Figure 1 and follow the following procedure: (1) preparing a homogeneous solution that contains a mixture of polymer and nanomaterial at a specific concentration; (2) blowing a bubble throw a nitrogen flow using a circular die by controlling some parameters, including pressure, expansion rate and external vertical force; and (3) depositing the bubble thin films onto solid or flexible substrates such as a silicon wafer and a plastic sheet. To clarify the key steps further, a schematic diagram for the BBFs’ method is shown in Figure 2a. In more detail, the nitrogen flow pressure is about (*P* = 150–200 Kpa), which allows the bubble to be expanded vertically at a rate of 10–15 cm ${min}^{-1}$. The bubble is produced with a diameter of ~35 cm and a height of ~50 cm. To obtain a larger bubble in diameter and height, a larger die and better control of the blown bubble must be applied. The trapped gas during the film transfer might affect the uniformity of the film, thereby generating a film defect. Controlling the parameters allows the bubble to be expanded in a vertical direction, which makes the nanomaterial orientation follow the upward direction. Hence, a good alignment of nanomaterial over a large area was obtained. The BBFs’ method has the potential to form uniform nanomaterial thin films with well-aligned, controlled density structures and scaled structures over a large area, leading to a 200 mm silicon wafer and 225 mm × 300 mm, which would pave the way for many real-life applications. Finally, the film thickness was changed by controlling the suspension volume and the final size of the bubble. 

The second technique was developed by Wu et al. in 2014 [52], who used similar steps to those of the first technique but with another polymer matrix: polymethylmethacrylate (PMMA). The bubble solution involved a mixture of PMMA (9.6 g, Mw ≈ 996,000) and 80 mL of acetone, which were then ultrasonicated for 30 min. A small quantity of nanomaterial was added to the bubble solution and stirred under magnetic stirring for 1 h until it became homogeneous. The bubble thin films were generated by (1) preparing a homogeneous solution contains a mixture of PMMA polymer and nanomaterial at a specific concentration; (2) blowing a bubble manually in air using a small portable tool and expanding it upward along the long axis; and (3) depositing the bubble thin films onto a substrate by lowering down the substrate towards film until touching it. The bubble was produced with a diameter of ~9 cm and a height of ~15 cm [52].

The third technique was developed by Wu et al. [53], who used steps similar to those of the first and second techniques but with another polymer matrix, namely, additive (F-127). More specifically, a concentration of 6 mg/mL of GO sheets was added to the bubble solution, which consisted of a mixture of F-127 and 30 mL of deionized water. It was then sonicated for 30 min. The bubble thin films were made manually by blowing the solution using air to form a thin layer of nanomaterials (GO) with a different surface coverage. The surface coverage increased by increasing the concentration of nanomaterial being embedded in the polymer film. For example, a study conducted by Wu et al. in 2015 [53] reported that the blown F-127 bubble onto Cu foil substrate to obtain a reduced oxide (RGO)–carbon hybrid film formed separated RGO sheets without much overlapping and a low concentration. As the GO sheets’ concentration increases, the surface coverage of RGO sheets increases. Also, RGO continuous sheets are obtained by an assembly process (layer-by-layer). Therefore, the surface coverage of nanomaterial increases with increasing it in the bubble solution.

**Figure 3:**
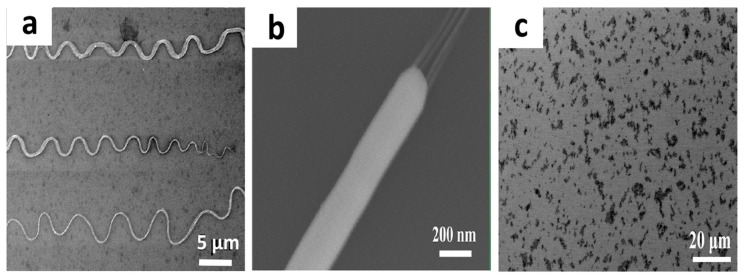
Scanning electron microscopy (SEM) images of different nanomaterial thin films fabricated using the BBFs’ method. Image (**a**) shows a SEM image of a nanospring array of tellurium nanowires (Te NWs) (5 µm scale bar). Adapted with permission from Ref [52]. 2014, American Chemical Society. Image (**b**) shows sausage-like nanostructures of graphene nanotubes filled by copper nanoblocks (GNT@CuNB) (200 nm scale bar). Adapted with permission from Ref [55]. 2016, American Chemical Society. Image (**c**) is an SEM image of graphene oxide sheets film (20 µm scale bar). Adapted with permission from Ref [53]. 2016, American Chemical Society.

The BBFs’ method is useful for the fabrication of nanomaterial thin films onto different types of substrates, along with high density and cross-junction structures at different configurations such as single- and double-layer nanomaterial arrays [52,54,55] and buckled nanospring nanomaterial arrays [52]. To be more precise, various types of nanomaterials are deposited onto a substrate using the BBFs’ method: single-wall carbon nanotubes (SWCNTs) [42], multiwall carbon nanotubes (MWCNTs) [42,54], cadmium sulfide nanowires (CdS NWs) [42], silicon nanowires (Si NWs) [42], tellurium nanowires (Te NWs) [52], and graphene oxide (GO) [53], as shown in Figure 3. This indicates that BBFs have been used for alignment of one-dimensional nanowires and nanotubes and two-dimensional sheets but have not yet been applied to zero-dimensional nanoparticles [42,52,53,54]. The quantity of those nanomaterials needs to be optimized when they are added to a polymer solution to control their organization and density. This is because each type of nanomaterial has an optimum concentration relative to that of the PMMA polymer. For example, the appropriate amount of nanomaterials added to the polymer solution for Te NWs was ~0.2 g [52], that for MWCNT was 0.0375 g [54], those for CdS NWs and Si NWs were 1–15 mg [42], and that for GO sheets were 6 mg/mL [53]. To make those nanomaterial thin films suitable for practical applications, they must be deposited onto a substrate. For this reason, many researchers have successfully deposited such nanomaterials onto different types of surfaces using the BBFs’ method, including Cu foil substrate [55], conventional Si wafer [52,54,55], quartz sheet [54], and a large piece of plastic sheet [52]. The bubble thin films deposited onto those surfaces consist of a single layer of aligned nanomaterial arrays. Multiple bubble thin films would result in many layers of nanomaterial arrays and hence increase the nanomaterials’ density [52]. Also, multiple bubble thin films deposited along different directions produce cross-junction structures [52,54,55].

According to the literature, the PMMA polymer is more favorable compared to the polymer F-127 and the polymer epoxy resin during the bubble thin films’ formation due to its advantages after thermal annealing. In more detail, the PMMA is used for two purposes. Firstly, it is used to blow bubbles containing nanomaterials to produce a network structure. Secondly, it serves as a sufficient carbon source for growing graphene, thus converting the film into graphene to produce a hybrid structure [54]. The annealing of the PMMA matrix on a substrate produced a clean nanomaterial or produced graphene films embedded with the chosen nanomaterial (i.e., a hybrid structure). These two properties are achieved by two parameters: the type of substrate and the annealing temperature. Precisely, PMMA-Cu nanowire films onto a Si substrate were annealed at 900 °C for 15 min, resulting in sausage-like carbon nanotubes@Cu nanoblock materials. In contrast, a similar sample was annealed at 1000 °C, but it resulted in ethe vaporation of such material [55]. Interestingly, PMMA-CNTs films onto a Cu foil substrate were thermally annealed at 1000 °C under H_2_/Ar flow in CVD furnace, giving a CNT-graphene film hybridized structure. However, a similar film was deposited onto a Si wafer and quartz sheet, which was thermally annealed at 700 °C, Ar atmosphere, resulting in the evaporation of the PMMA matrix, leaving a clean CNT network [54]. More interestingly, similar results were obtained using the F-127 polymer in a study carried out by Wu et al. in 2015 [53]. To elaborate, the annealing of the deposited F-127 film onto Cu foil resulted in a continuous carbon film (annealing at 1000 °C for 15 min, H_2_/Ar atmosphere). In contrast, the annealing of the deposited F-127 film onto the Si wafer resulted in the evaporation of the film. This means that Cu foil substrate is critical for obtaining the carbon film. More importantly, the PMMA and F-127 polymers both acted as carbon sources for growing carbon film (graphene). This is very important since the polymer films have solved the two most common issues in the assembly of GO sheets in the form of a thin film, including the aggregation of GO and the presence of boundaries between the sheets, which limited its application in many areas such as thin-film devices, as reported by Wu et al. in 2015 [53]. Finally, the PMMA embedded with nanomaterials results in low-performance device application, and hence it must be removed. Its removal process involves a wash using an organic solvent (acetone) at room temperature as follows: (1) supposing one has deposited the film onto a silicon substrate, it then covers the other side of the silicon substrate; (2) thus, the two Si sides need to clamped by a magnet and immersed in acetone for three days to dissolve the PMMA, leaving the nanomaterials on the substrate [52].

The BBFs’ method has had several applications, including transistors, photodetectors, gas sensors, solar cells, and electrical measurements, proving its efficiency, consistency, scalability, and quality control. Precisely, for transistor applications, the Si nanowire bubble thin films were transferred to a plastic substrate to be used for field-effect transistor (NW-FETs). Such transistors were fabricated as three repeating transistor arrays, each one consisting of 400 multi-NW transistors. These transistors contain a number of NWs per device and their typical I –V characteristics show the device’s uniformity characteristics, with “yield a peak transconductance, gm = ¼ dI_d_/dV_g_, of 6 µS with an on current, *I*_on_, of ~16 µA, an on/off ratio ˃10^5^ and a threshold voltage, Vt, of 0.55 V” [42]. The fabrication of such a device was reproducible, made with high density, good alignment, and high distribution of the NWs [42].

The BBFs’ method has also been used for nanoelectronic devices applications such as photodetectors and gas sensors. Wu et al. in 2014 [52] fabricated a device consisting of two Au electrodes patterned into a silicon surface and perpendicular to the aligned Te NWs. The electrical properties of such a device was measured, where the current–voltage curve indicated a resistance of 0.5 MΩ, a current of ∼2.4 μA (dark), and a current of ∼4.5 μA (light) under 45 mW/cm^2^ illumination. Although the numbers of NWs are different, their behavior is stable since the light-to-dark current ratio (I_L_/I_D_) is stabilized within the range between 1.5 and 1.8. Another important application was a gas sensor using Te NWs, which indicated a good response to NO_2_ down to a concentration of 10 ppm. The device time-domain measurements showed a fast response after introducing NO_2_ with reproducible adsorption/desorption behavior.

**Figure 4:**
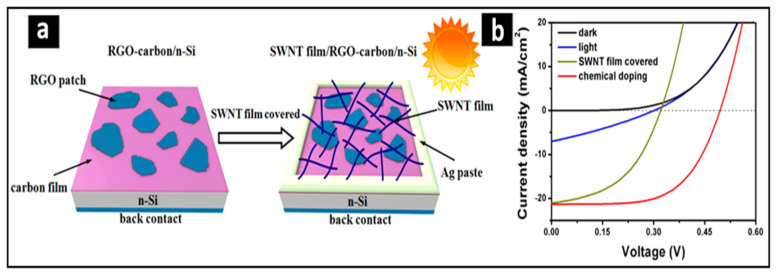
Fabrication of solar cell device using the BBFs’ method (**a**) Models of the RGO–carbon/Si solar cell structure. The carbon film, RGO film, and CNT film are in contact with the top of the silicon wafer. While gallium–indium was applied to the back side of the Si wafer, Ag was pasted around such a film. (**b**) J−V curves of the solar cells in dark and light conditions under AM 1.5. Adapted with permission from Ref. [53]. 2015, American Chemical Society.

Another useful application was solar cells, which are crucial for a sustainable energy future. Wu et al., in 2015 [53], fabricated a solar device that combined RGO−carbon hybrid films with crystalline Si. In more detail, the carbon film was in contact with the silicon wafer, followed by the RGO film and then the CNT film. Gallium–indium was applied to the back side of the silicon as the back electrode and Ag was pasted around the film to define active light reception. The RGO–carbon hybrid films functioned as transparent electrodes, making effective p−n junctions with Si and transporting holes to the external circuit. The SWNT film acts as the hole–transport layer in the active area (Figure 4a). This study concluded that adding an RGO film on top of a carbon film enhanced the short-circuit density (J_sc_) of the cell from 3.22 to 6.94 mA/cm^2^, improved the diode characteristic, and reduced its carrier combination. Adding the CNTs to the RGO-carbon films increased the J_sc_ of the cell from 6.94 to 21.03 mA/cm^2^ with a cell efficiency of 3.17%. Finally, the cell efficiency increased further to 6.42% after performing chemical doping to the device with HNO_3_ treatment (Figure 4b).

Another study was conducted by Wu et al. in 2015 [54] to fabricate heterojunction solar cells consisting of multi-walled carbon nanotubes (MWCNTs) and graphene hybrid film. It was found that MWCNTs/n-Si solar cells resulted in very low conductivity with poor cell efficiency of 0.73%. Cell efficiency increased after hybridization of graphene with MWCNTs using the BBFs’ method (cell efficiency of 1.26%). Cell performance increased further after coating with titanium dioxide TiO_2_, chemical doping with HNO_3_ and H_2_O_2_ treatment with an efficiency of 9.24%. The study concluded that a MWCNT–graphene hybrid synthesized by the BBFs’ method can be used in applications as transparent conductive electrodes. Further possible applications will be discussed in the Discussion section below.

## 3. Bubble Deposition Method (BDM)

The bubble deposition method was first developed in 2006 by Benattar et al. [49] when a solution of nonionic surfactant (C_12_ E_6_) in ultrapure water was prepared at a critical micelle concentration (CMC). After that, a silica disc was immersed in such a solution, and a bubble was formed at the top of the disc by injecting air through it. After forming the bubble, a hydrophobic silicon substrate was brought into contact with the bubble surface to fully transfer the film. Another study was also conducted by Benattar et al. in 2008 [57] to investigate the film formation of two types of surfactants, including (C_16_F_13_SOTHAM) and (C_12_ E_6_). It also examined the effect of their bilayer transferred into hydrophilic and hydrophobic silicon and glass substrates. Other studies have used ionic, nonionic and cationic surfactants, which are deposited onto hydrophobic and hydrophilic silicon and glass substrates [58,59,60]. These relevant studies indicated that most of the ionic surfactants result in a well-assembled structure, while the nonionic and cationic surfactant types result in a poorly assembled structure [49,58,59,60]. To date, the ionic surfactant Sodium Dodecyl Benzene Sulphonate (SDBS) is the only type that has successfully been used for forming nanomaterial thin films using the BDM. In the following sections, the BDM will be discussed in more detail.

**Figure 5:**
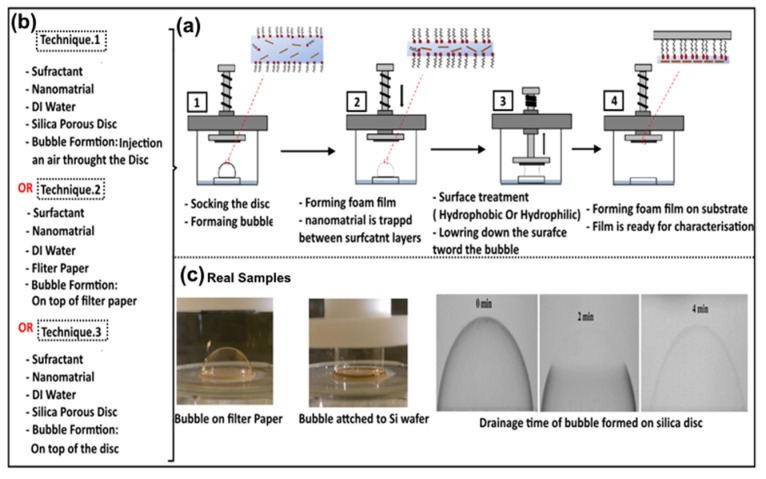
Schematic representation of the bubble deposition method (BDM). (**a**) A diagram illustrating the four key stages utilized to fabricate the thin film, which are (1) forming a bubble, (2) forming a foam film, (3) surface treatment, (4) film deposition onto a substrate. Adapted with permission from Ref [61]. 2015, American Chemical Society (**b**) The three main techniques which have been applied to perform the first key stage (i.e., forming bubble) (in the left side of the figure). (**c**) Some of the real images taken of the bubble during its formation stages. Adapted with permission from Ref [60]. 2015, American Chemical Society. Adapted with permission from Ref [61]. 2017, American Chemical Society.

The relevant literature indicated that thin foam films using the BDM can be formed through three main techniques. The first technique was put forward by Benattar et al. (2006) [49]. The bubble was formed on top of a porous silica disc by passing air through a hole already made at the bottom of the disc. After a few minutes, the bubble started to drain, and a change in the film color was observed, indicating that the foam film had been formed. When the whole bubble surface was completely drained, a hydrophobic silicon substrate was brought into contact with the top of the bubble surface. As a result, the foam film was transferred into the solid surface and became ready for characterization. The second technique was also proposed by Benatter et al. in 2008 [57], when they used a filter paper instead of a porous silica disc, and the bubble was formed on top of the filter by a glass pipette. The third technique was developed by Alshehri et al. in 2015 [59], when they combined the previous two techniques—i.e., the bubble was formed on top of the silica porous discs using the glass pipette—in order to suggest a more reliable and simpler approach. The experimental process of forming bubble thin films is shown in Figure 5. It summarizes the three techniques and the stages of bubble thin film formation, and shows real bubble thin film images. To further clarify the key steps, a schematic diagram for the BDM is shown in Figure 2b.

The purpose of using either the porous silica disc or the filter paper material is to control the drainage process and time [49,59,61]. In the case of the porous silica disc, it must be soaked in the surfactant solution up to half of it; otherwise, the bubble will not be stable, and bubble drainage will become slow. The disc is reusable and can cleaned by placing it in a mixture of DI water and sulfuric acid for 20–30 min and then boiling it in DI water for 20–30 min to extract the acid and finally drying in for 1.5–2 h at 200 °C or 250 °C (note: any remaining acid inside the disc will affect film formation and stability) [59]. Before bubble formation, the filter paper needs to be rinsed in boiling ultrapure water because it should be wet enough to make a stable bubble; otherwise, film drainage will become slow and the bubble might burst [57].

The bubble thin films can be deposited as a pure surfactant or as nanoparticles embedded within the bilayer of the surfactant. Such films are deposited onto different types of substrates, including silicon wafer [59,60], glass [57], polyamide and plastic [62]. These substrates will be discussed in turn below. The first substrate is a silicon wafer, which can be changed to be hydrophobic or hydrophilic. To be hydrophobic, two methods are used: (1) wet chemical etching and (2) salinization using octadecyltrichlorosilane (OTS). In the case of wet chemical etching, the surface is immersed in a buffered oxide etch (BOE) for a few minutes, followed by rinsing with acetone, ethanol, water, and finally drying with either air or nitrogen gas [2]. The BOE is a mixture of a buffering agent, ammonium fluoride (NH_4_F), and hydrogen fluoride (HF) (note: HF is a dangerous solution) [63]. This process results in a contact angle of 90°, indicating hydrophobicity properties of the silicon surface. In the case of salinization using octadecyltrichlorosilane (OTS), in contrast, the silicon wafer is immersed in a solution containing 5 mmol/L OTS in 70% chloroform and 30% hexadecane. This solution must be poured into a beaker, which is placed in an ultrasonic bath, at a reaction temperature of 20 °C for a reaction time of 6 min. After that, the substrate is washed with chloroform and dried with nitrogen [57]. However, the use of HF acid has significant health and environmental concerns, such as corrosive effects, respiratory issues, water pollution, and air quality. It requires training with proper safety protocols to be used carefully under management and regulation. Although the salinization process using octadecyltrichlorosilane (OTS) takes more time compared to the wet chemical etching by HF, it is more recommended to be used to avoid environmental and health concerns caused by HF. To make the silicon surface hydrophilic, it needs to be exposed to UV-ozone for approximately 10 min. This treatment yields a contact angle of 20°, which indicates the hydrophilicity properties of the silicon surface [64]. It should be noted that before controlling the silicon properties, it must undergo two steps of treatment. Firstly, the substrate is cleaned with acetone, ethanol, and water in an ultrasonic bath for 5 min in each solvent separately. Secondly, the cleaned substrate is placed in a piranha solution for 30 min, to eliminate any organic contaminants, and then rinsed thoroughly with deionized water and dried with air or nitrogen. The piranha solution consists of a mixture of sulfuric acid (H_2_SO_4_) and hydrogen peroxide (H_2_O_2_) (ratio 3:1) [58]. The second substrate is glass, which is cleaned with acetone, ethanol, and water in an ultrasonic bath for 5 min in each solvent separately. The cleaned substrate is then placed in a piranha solution for 30 min to remove any organic contaminants and then rinsed thoroughly with deionized water and dried with air or nitrogen [58]. After that, the substrate is immersed in a solution involving 5 mmol/L OTS in 70% chloroform and 30% toluene. This solution must be poured into a beaker, which is placed in an ultrasonic bath, at a reaction temperature of 20 °C for a reaction time of 6 min. Finally, the substrate is washed with chloroform and dried with nitrogen [57].

The surfactant foam film is usually formed and then transferred onto hydrophobic or hydrophilic solid surfaces to become suitable for practical applications. For this reason, several studies have investigated the transferred properties of such surfactants, considering their types (i.e., nonionic, cation and ionic). For example, nonionic surfactants such as hexaethylene glycol monododecyl ether (C_12_ E_6_) and fluorinated sulfoxide (C_16_F_13_SOTHAM) have formed a well-assembled structure on hydrophobic glass and Si surfaces [58]. However, the nonionic surfactant, such as Dodecyldimethylpho-sphine oxide (C_12_DMPO), has formed a poorly assembled structure after transferring it onto both hydrophobic and hydrophilic surfaces [60]. With respect to the cationic surfactant, a poorly assembled structure was developed with Cetyltrimethylammo-nium bromide (CTAB) when it was deposited onto the Si hydrophobic surface. The last type is the ionic surfactant, which has formed a well-self-assembled structure on a hydrophobic solid surface such as Sodium Dodecyl Benzene Sulphonate (SDBS) [56,61,62] and hexadecyltrimethylammonium bromide (C_16_TAB) [60].

**Figure 6:**
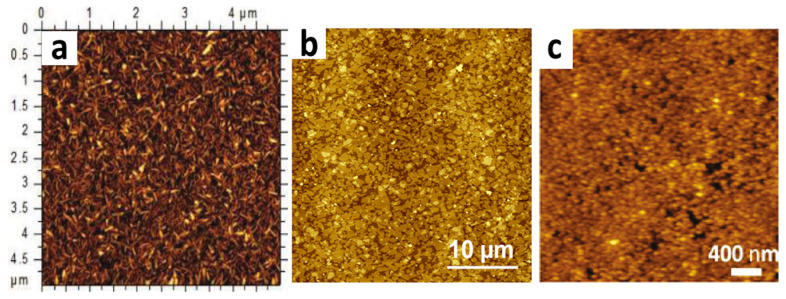
Atomic force microscopy (AFM) topography images for different types of nanomaterial thin films deposited onto different types of substrates using the BDM. (**a**) AFM topography image of CeVO_4_ NW-SDBS film deposited onto silicon substrate. Adapted with permission from Ref [64]. 2011, American Chemical Society. (**b**) AFM topography image of small hydrophilic GO flakes deposited onto a quartz substrate. Adapted with permission from Ref [61]. 2015, American Chemical Society. (**c**) AFM topography image of a silica nanoparticles film deposited onto a silicon substrate. Adapted with permission from Ref [56]. 2010, American Chemical Society.

To make pure surfactant thin films suitable for practical applications, it is necessary to find a way to embed nanomaterials in their bilayer and deposit them onto the solid substrate. Several studies have successfully embedded nanomaterials within the surfactants’ bilayer with a large-scale assembly to be used in many applications. For example, Tang et al., 2010 [62] have assembled single-wall carbon nanotubes (SWCNTs) on different types of substrates, including silicon wafer Si (111), polyethylene terephthalate (PET), and Kapton (Polyimide Film) with highly aligned and densely packed nanomaterials. Other studies have also employed different types of nanomaterials such as silica nanoparticles [56], CeVo_4_ [64], gold nanoparticles [65], graphene oxide [66], and multilabel nanomaterials (SWCNTs, CeVO_4_ NWs, and silica NP). An example is shown in Figure 6 [67]. All of such nanomaterials were deposited on a silicon wafer Si (111) and glass. Moreover, the graphene oxide was deposited onto polyimide substrate and a quartz wafer. According to the relevant body of scholarship, such nanomaterials were preferred to be embedded within the bilayer of an ionic surfactant. However, they were not embedded within the bilayer of both a cationic and a nonionic surfactant type since they did not form a well-self-assembled structure onto the solid surface. To date, the only ionic surfactant that has been widely used to make nanomaterial thin films is SDBS due to its well-self-assembled properties onto different types of substrates and ease of elimination after deposition. Another ionic surfactant that formed well-self-assembled properties is C_16_TAB. However, it has not been used for assembling nanomaterials, thus needing further research studies. All of the above studies have demonstrated the reproducibility of the BDM with any type of nanomaterial with a good thin film organization at a high density and located on different geometric structures.

The main parameters that control the self-assembled of nanomaterials onto the solid substrates include surfactant concentration, nanomaterial concentration, deposition drainage time, and surface wettability (i.e., surface energy). More precisely, the homogeneous film surfactant is formed at any CMC surfactant concentration. However, it is not any nanomaterial concentration that can be used with any CMC surfactant concentration to make a thin film of such nanomaterials. Thus, a film solution (i.e., homogenous surfactant concentration with nanomaterials’ concentration into a solvent) must be selected properly, where the nanomaterials’ concentration is relative to the surfactant concentration, because an excessive concentration of surfactants could negatively impact the organization or alignment of the nanomaterials involved [56,65].

The deposition drainage time refers to the time required for the bubble to be drained before deposition. It depends on the type of surfactant and the solvent chosen. For example, the SDBS surfactant with water as a solvent requires 4 min to be completely drained, while a 1-palmitoyl-2-oleoyl-sn-glycero-3-phosphocholine (POPC) molecule with formamide (FA) as a solvent requires 20 min [59,62]. The FA requires a longer lifetime to form foam films compared to that film used in an aqueous solution due to its low vapor pressure [59]. The nanomaterials should not always be embedded at a completely drained stage but could be embedded at any drainage time. For example, two nanomaterials are embedded with SDBS surfactants at different drainage times: carbon nanotubes (CNTs) at 2–3 min [62] and silica nanoparticles at 1 min [56]. Thus, it is necessary to optimize and find out the drainage time that meets the relative concentrations of surfactants and nanomaterials. Otherwise, the nanomaterials would be lost from the film surface, and hence no materials would be deposited onto the substrate.

**Figure 7:**
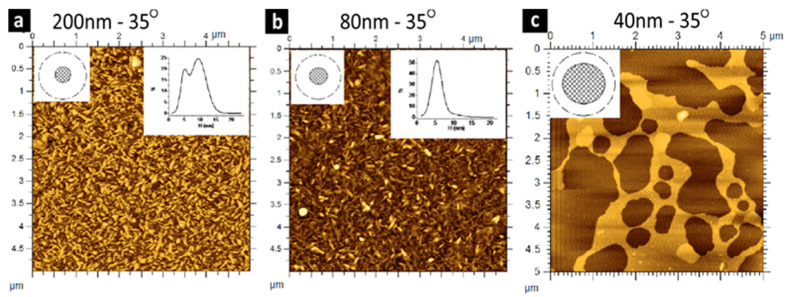
Atomic force microscopy (AFM) topography images of CeVO_4_ nanowire thin films fabricated using the BDM. Those three samples were made using SDBS surfactant at three different bubble drainage times of (**a**) 200 nm, (**b**) 80 nm, and (**c**) 40 nm, keeping the surface contact angle constant at 35°. These three images represent the effect of drainage time on the density of the nanomaterials. Adapted with permission from Ref [64]. 2011, American Chemical Society.

Drainage time (film ripening) is one of the key parameters influencing both the high density and homogeneity of the nanomaterials [64]. Drainage time affects the density of the nanomaterials. For example, Costa-Coquelard et al., in 2011 [64], studied the impact of three different film thickness (200 nm, 80 nm, 40 nm) on controlling the density and surface coverage of CeVO_4_ nanowire. The AFM measurements of that study indicated that the solid surface for both the 200 nm and 80 nm films was 100% entirely covered, but with more monolayer at 80 nm film, as shown in Figure 7a,b. For the 40 nm film, it was inhomogeneous with low surface coverage, as shown in Figure 7c. These results prove the impact of drainage time on nanomaterial surface coverage.

**Figure 8:**
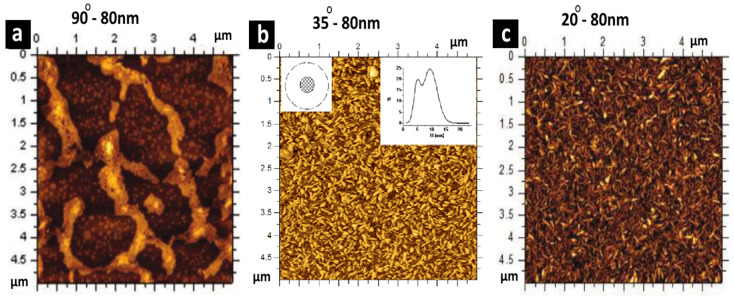
Atomic force microscopy (AFM) topography image of CeVO_4_ nanowire thin films fabricated using the BDM. These films were made using SDBS surfactant at three different surface contact angles of (**a**) 90°, (**b**) 35°, and (**c**) 20° while keeping the drainage time constant at a bubble thickness of 80 nm. These three images represent the effect of the surface energy (surface wettability) on etch deposition and alignment of nanomaterials on a substrate. Adapted with permission from Ref [64]. 2011, American Chemical Society.

Surface energy (surface wettability) plays a significant role in the deposition and alignment of nanomaterials on a substrate. For example, Costa-Coquelard et al., in 2011, [64] studied the influence of a hydrophobic surface (90°) and hydrophilic surfaces (35° and 20°) on controlling the density and surface coverage of a CeVO_4_ nanowire while keeping the film thickness at 80 nm. The AFM data suggested that inhomogeneous films containing NWs aggregated were observed at 90°. However, the film deposited at 35° formed a monolayer of NWs with defects on the surface. When the film was deposited with a more hydrophilic surface at 20°, the surface was entirely covered by homogeneous monolayers of NW, as shown in Figure 8a–c. Therefore, increasing the surface wettability controlled the film structure favorably.

To date, relevant studies that have used the BDM prove that the method is suitable and reliable for transferring many types of nanomaterial thin films onto different types of solid substrates with different geometric structures. However, there are still limited practical applications for the BDM. Only one study conducted by Azevedo et al. in 2015 [61] is presented here, in which the authors successfully used a reduced graphene oxide (rGO) as transparent electrodes with tuned transparencies and conductivities. That study indicated that the reduction made under acetylene atmosphere showed satisfactory performance for prepared rGO electrodes compared to other studies. Therefore, further studies are required in terms of potential applications of the BDM. Some of the suggested applications will be discussed in the Discussion section below.

## 4. Discussion

This review paper describes and discusses two main important methods for making nanomaterial thin films: the bubble blown films’ (BBFs) method and the bubble deposition method (BDM). The BBFs’ method has been used for organizing a wide range of nanomaterials, which were only one-dimensional and two-dimensional, such as SWCNTs, MWCNTs, CdS NWs, Si NWs, Te NWs, and GO sheets. These nanomaterials were deposited onto solid surfaces such as Cu foil, silicon wafer, glass plastic sheet, and quartz sheet. However, zero-dimensional nanomaterials have not been used in any previous studies. According to the method principle, zero-dimensional nanomaterials can be assembled onto substrates [68,69] due to the shear stress on the bubble solution during its blown process, which produces space between all the classifications of nanomaterials after depositing a single bubble [70]. Nevertheless, despite this space, the nanomaterials are still useful for many applications such as solar cells [71], sensors [72], transistors [73], nanoelectronics devices [74], and semiconductor applications [75].

A number of studies [52,53,54] have been carried out in an attempt to address some of the BBFs’ method issues around the surface coverage, film thickness, and density structure. They specifically investigated the impact of nanomaterial thin films parameters such as nanomaterials’ concentration, suspension volume, and the final size of the bubble. However, there are some unresolved issues associated with the defect control of large areas of the bubble, such as controlling the space between nanomaterials with high density to define interconnections using only one blown bubble at once. This might be related to control over nitrogen pressure flow, bubble expansion rate, control of external vertical force, and more. Therefore, it is recommended to refine surface chemistry further to obtain a single bubble containing a high quantity of nanomaterials with limited space between them. This refinement is useful in offering good compatibility with the polymer industry.

There are some main properties of the BBFs’ method. For example, it produces 3D nanomaterial thin films with a network structure including NWs, NTs, and graphene sheets. It also produces a high density of nanomaterials using the PMMA polymer, but after multiple bubble depositions. The PMMA works as a sufficient carbon source to grow a graphene structure, which offers an opportunity to produce a composite or hybrid nanostructure. In addition, it is a low-cost and highly scalable method because it allows for the formation bubbles in large, flexible substrates, with a diameter of 35 cm using a die and 9 cm using PMMA [52,53,54].

The bubble deposition method (BDM), in contrast, has been used to fabricate thin films of nanomaterials such as assembled single-wall carbon nanotubes (SWCNTs), silica nanoparticles, CeVo_4_, gold nanoparticles, and graphene oxide. These nanomaterials were deposited onto different types of substrates such as silicon wafer Si (111), polyethylene terephthalate (PET), and Kapton (Polyimide Film). Such nanomaterial thin films were mainly produced by surfactants that generated a foam film structure. These surfactants are different in their types, and relevant studies [57,60,62] demonstrated that only the ionic surfactant type could form a thin film with nanomaterials because it formed a well-assembled structure on the substrates. These studies also showed that the only surfactants that contained this structure were Sodium Dodecyl Benzene Sulphonate (SDBS) and hexadecyltrimethylammonium bromide (C_16_TAB). However, to my knowledge, the SDBS surfactant has been used to make a thin film with nanomaterials, but the C_16_TAB surfactant has not been used with any nanomaterials. Thus, it is recommended to conduct further studies in order to determine what nanomaterials can be used with the C_16_TAB surfactant. Further studies also need to be carried out to explore whether all ionic surfactants can form a well-assembled structure on the substrates and make nanomaterial thin films.

**Figure 9:**
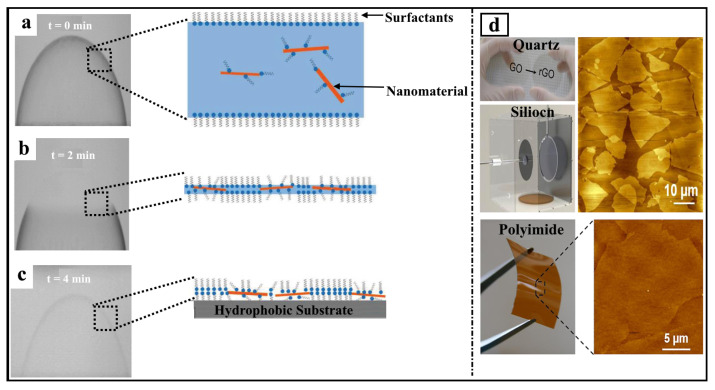
Schematic representation of a mechanism of nanomaterial thin films made by the BDM. (**a**) A photograph of bubble thin films formed at t = 0 min, showing a sketch of the liquid layer, which forms an interface with air, which is dominated by rich surfactants. (**b**) A photograph of a bubble formed at t = 2 min after drainage of the bubble surface, showing a sketch of liquid layer evaporation. (**c**) A photograph of a bubble formed at t = 4 min after full drainage of the bubble surface, which is deposited onto the hydrophobic substrate, showing the insertion of nanomaterials between the surfactant monolayers to form a film. Adapted with permission from Ref [60]. 2017, American Chemical Society. (**d**) AFM images of GO flakes transferred onto a quartz substrate, a silicon substrate, and a polyimide substrate. Adapted with permission from Ref [61]. 2015, American Chemical Society.

For further clarification, the mechanism of nanomaterial thin films’ formation will be discussed below. After forming the bubble (i.e., t = 0 min), a liquid layer forms an interface with air, which is dominated by rich surfactants (Figure 9a). Over time (i.e., t = 2 to t = 4 min), the liquid layer evaporates, resulting in an increase in the ratio of the surface area to volume (Figure 9b,c). The surfactants interact with each other to form monolayers. After that, the nanomaterial starts to be trapped between the surfactant’s monolayers close to the hydrophilic head of the surfactants (the surfactants work as nanomaterial carriers) (Figure 9b). Such surfactant molecules with trapped nanomaterials interact with a substrate with a large degree of orientation, with a hydrophobic chain pointed toward the surface (Figure 9c). Therefore, nanomaterials are self-assembled onto the substrate, forming nanostructured thin films [56,59,60,67]. The self-assembled nanomaterials are controlled by many parameters, such as surfactant concentration, nanomaterial concentration, deposition drainage time, and surface wettability. These parameters have been discussed in detail in the previous section. Such a mechanism demonstrated that the surface of the surfactant solution and the interface between the surfactant layer with the substrates are ordered and formed separately. Accordingly, the structure of the foam film that carried nanomaterials occurred before the surfactants interacted with the substrate. This process took place due to several factors, such as the interactions between solute molecules, between solute and solvent molecules, and between solute molecules and the substrates.

The BDM has a number of key features. Firstly, it produces 2D nanomaterial thin films with a network structure. Secondly, after depositing only one bubble, it provides a high density of nanomaterial thin films using the ionic SDBS surfactant, which is easy to remove. Thirdly, it is a low-cost and scalable method for forming thin films since it is used for organizing two-dimensional GO and RGO nanomaterials on positions 2 and 8 in the silicon wafer, as shown in Figure 9d [61]. The BDM also has some limitations. First, it uses hydrogen fluoride (HF) chemicals during the experimental procedure, which is dangerous. Also, whilst there were several studies conducted to organize the surfactants and nanomaterials onto different types of solid substrates [58,59,60], they have not been able to demonstrate their potential applications. Finally, a number of studies have been carried out in an attempt to address some of the BDM’s issues around surface coverage, film thickness, and density structure, as discussed in detail in the previous section. They specifically investigated the impact of nanomaterial thin films’ parameters, such as surfactant concentration, nanomaterial concentration, deposition drainage time, and surface wettability. However, there are unresolved issues that researchers are recommended to consider, including factors influencing the long-term stability of bubble-derived films, such as film composition additives (salts), environmental conditions (humidity and temperature), and mechanical properties (stress resistance and elasticity), which are essential for film swelling, dissolution, stretching and compression, allowing for the development of a long-term stabilized film and the optimization of proper applications. Table 1 below summarizes the advantages and disadvantages of the two nanostructured bubble thin films: BBFs and the BDM. It also compares a traditional method used for assembling nanomaterials onto a substrate (Langmuir–Blodgett method) with BBFs and BDM.

Nanomaterial thin films are promising candidates for many potential applications across different fields, such as environmental science and biomedicine. In the field of environmental science, nanomaterial thin films have been coated on membrane filtration to enhance its process and effectively remove contaminants, such as heavy metals, bacteria, and organic pollutants [79]. Other nanomaterials, such as cobalt oxide nanostructures, were used in gas sensors applications to detect air pollutants, which are dangerous to humans and the environment, such as hydrogen sulfide (H_2_S), nitrogen oxides (NO, NO_2_), [80] and sulfur dioxide (SO_2_) [81]. Furthermore, titanium dioxide (TiO_2_), in the form of a thin film, has been used for photocatalytic degradation of organic pollutants in water and air applications owing to its important combination of properties [82]. In the field of biomedicine, nanomaterials have been used for rapid detection of modes for a multitude of biomarkers related to diseases, giving the opportunity for early diagnoses [83]. More interestingly, they can be used to delay consecutive drug dosages for operation-time-controlled systems [84]. Also, nanomaterial thin films can be used to deliver drugs, supporting their fast delivery or long residence time. Finally, researchers revealed that nanomaterials have been functionalized to specific cells or tissues to reduce doses and side effects of therapies or systemically injected compounds [85].

To conclude, the BBFs’ method has used 1D and 2D nanomaterials; however, 0D nanomaterials have not been used in any previous study. Therefore, these nanomaterials need to be considered and used for further applications across different fields, such as biomedicine and environmental sciences. Also, there are some unresolved issues associated with the defect control of large areas of the bubble, such as controlling the space between nanomaterials with high density to define interconnections using only one blown bubble at once, which need to be explored further. The BDM, in contrast, has intensively used one ionic surfactant to form nanomaterial thin films: Sodium Dodecyl Benzene Sulphonate (SDBS). Thus, further studies need to be carried out to explore whether or not all ionic surfactants can form a well-assembled structure on the substrates and make nanomaterial thin films. In addition, there is an ionic surfactant which has not been used with any nanomaterials: hexadecyltrimethylammonium bromide (C_16_TAB). Therefore, it is recommended to undertake further studies in order to determine what nanomaterials can be implemented with it. Also, further studies need to be conducted to explore the ability of nonionic and cation surfactant types to form nanomaterial thin films, with further investigation of the main parameters, such as surfactant concentration, nanomaterial concentration, deposition drainage time, and surface wettability. Furthermore, researchers have to address the unresolved issues associated with the defect control of large areas of the bubble and conduct comprehensive studies focusing on the factors influencing the long-term stability of bubble-derived films. Filling these gaps would improve the method further in terms of cost and film quality and hence offer recommendations of more reliable applications. Finally, the BDM needs to be implemented in advanced applications, such as optoelectronics, sensors, energy storage, magnetic devices, and coatings.

## 5. Conclusions

This article has reviewed two important methods that are used for assembling nanomaterial thin films: the BBFs’ method and the BDM. It has outlined their history, fabrication techniques, key features, and applications. Most of the nanomaterials used with these two methods are suitable for many applications due to their high surface-area-to-volume ratio and unique properties. Both methods meet the requirements of depositing nanomaterial-based devices onto different types of solid and flexible substrates, including scalability, low-cost, reliability, fast operation speed, good alignment, high density, and uniformity. The most important applications for these methods are nanomaterial-based device applications, including transistors, sensors, solar cells, catalysts, heat dissipation, light-emitting devices, energy storage, and protective layers. The ability to combine the two methods’ key features with the unique properties of nanomaterials to make devices opens new avenues for technological innovations.

## Figures and Tables

**Table 1:** A summary of advantages and disadvantages of BBFs and the BDM comparing with the Langmuir–Blodgett method.

Comparison Criteria	Blown Bubble Films (BBFs)	Bubble Deposition Method (BDM)	Langmuir–Blodgett Method
Advantages	(1) The method assembles 3D nanomaterial thin films onto solid and flexible substrates.	(1) The method deposits 2D nanomaterial thin films onto solid and flexible substrates.	(1) The method is able to assemble an individual molecule into 2D and 3D systems [76].
	(2) The method allows for precise control over film thickness at the molecular level.	(2) The method allows for precise control over film thickness at the molecular level.	(2) The method enables precise control over the film’s thickness and arrangement and it is suitable for creating uniform layers and complex multilayer structures [77].
	(3) The method allows for organizing molecules in a specific orientation and it is localized.	(3) The method allows for organizing molecules in a specific orientation and it is localized.	(3) The method allows for organizing molecules in a specific orientation and it is localized.
	(4) The method is low-cost and highly scalable, making it suitable for industrial processes.	(4) The method is low-cost and highly scalable, making it suitable for industrial processes.	(4) The method allows different types of materials to create films, such as organic and inorganic compounds [76].
		(5) The method allows for making films with high-density materials at a fast operation speed with good alignment and arrangement to control the interparticle distances with only one bubble thin film’s deposition.	
		(6) The method is easy to operate and does not require hard equipment or control of environmental conditions.	
Disadvantages	(1) The method organizes the nanomaterials with controlled alignment over large areas, but controlling the space between nanomaterials with high density to define interconnections requires multiple deposited films.	(1) The method uses hydrogen fluoride (HF) chemicals during the experimental procedure, which is dangerous.	(1) The method is expensive, making it less accessible for some research and industrial applications [78].
	(2) The method is complex to operate and requires precise control of nitrogen pressure flow, bubble expansion rate and control of external vertical force.		(2) It remains uncertain whether this method can be used for scaling up nanomaterials films onto large solid and flexible substrates.
			(3) The method is complex and requires precise control of environmental conditions [78].
			(4) Monolayer film structures are often modified, making it difficult to obtain a high-quality film [78].
			(5) Film defects are possibly produced during their transfer process, especially for softer or unstable molecular films [77].

## Data Availability

Not applicable.

## Abbreviations

The following abbreviations are used in this manuscript:

BBFs	bubble blown films
BDM	bubble deposition method
(NBFs)	Newton black films
CBFs	common black films
PMMA	poly (methylmethacrylate) polymer
Te NWs	tellurium nanowires
RGO	reduced graphene oxide
CNTs	carbon nanotubes
GO	graphene oxide
SWCNTs	single-wall carbon nanotubes
MWCNTs	multiwall carbon nanotubes
CdS NWs	cadmium sulfide nanowires
Si NWs	silicon nanowires
Te NWs	tellurium nanowires
NW-FETs	nanowire-filled effect transistor
CMC	critical micelle concentration
SDBS	sodium dodecyl benzene sulphonate
BOE	buffered oxide etch
HF	hydrogen fluoride
(NH_4_F)	ammonium fluoride
(H_2_SO_4_)	sulfuric acid
H_2_O_2_	hydrogen peroxide
C_12_DMPO	fluorinated sulfoxide (C_16_F_13_SOTHAM)
CTAB	Dodecyldimethylpho-sphine oxide
(C_16_TAB)	hexadecyltrimethylammonium bromide
AFM	Atomic Force Microscope
SEM	Secanning electrcon micrsope
